# Citizen eyes on elusive wildlife: Assessing public appreciation for urban wild mammals

**DOI:** 10.1007/s13280-025-02315-5

**Published:** 2025-12-09

**Authors:** Emiliano Mori, Valentina Marchi, Olivia Dondina, Andrea Viviano, Pietro Di Bari, Rosario Balestrieri, Marida Corradetti, Leonardo Ancillotto

**Affiliations:** 1 Research Institute on Terrestrial Ecosystems (IRET), National Research Council of Italy (CNR), Via Madonna del Piano 10, 50019 Sesto Fiorentino, Florence, Italy; 2 National Biodiversity Future Center (NBFC), Piazza Marina 61, 90133 Palermo, Italy; 3 National Research Council of Italy - Institute of BioEconomy (CNR-IBE), Via G. Caproni 8, 50145 Florence, Italy; 4 Department of Earth and Environmental Sciences, University of Milano-Bicocca, Piazza Della Scienza 1, 20126 Milan, Italy; 5 Department of Earth and Marine Sciences, University of Palermo, Via Archirafi 22, 90123 Palermo, Italy; 6 Department of Integrative Marine Ecology, Stazione Zoologica Anton Dohrn, CRIMAC, Calabria Marine Centre, Contrada Torre Spaccata, Amendolara, Cosenza, Italy; 7 Istituto Istruzione Superiore Agrario Celso Ulpiani, Via Della Repubblica 30, 63100 Ascoli Piceno, Italy

**Keywords:** Familiarity effect, Human-wildlife coexistence, Social perception, Urban ecology, Urban wildlife

## Abstract

Urbanisation is reshaping ecosystems, posing both challenges and opportunities for biodiversity conservation, particularly for urban mammals. We investigated public appreciation of 15 wild mammal species occurring in cities through a visual survey of 1000 respondents across ten Italian cities. Participants ranked species by desirability in urban environments. Hedgehogs, squirrels, rabbits, and roe deer were consistently preferred, possibly for their broad urban presence, whereas rats, wild boar, and coypus were less positively rated, possibly because of the widespread fears of health risks, damage, or invasiveness. Wolves elicited polarised reactions, possibly reflecting complex cultural meanings, including fascination and human-wildlife conflict issues. Geographic variation was marked: some species were preferred where familiar (“familiarity effect”), whereas large carnivores were highly valued where absent (“distance effect”). Visual survey tools effectively captured instinctive responses. Results stress the need for localised, species-specific education and conservation strategies that integrate citizen-science to improve coexistence in urban landscapes.

**Supplementary Information:**

The online version contains supplementary material available at 10.1007/s13280-025-02315-5.

## Introduction

Urbanisation is one of the dominant global trends reshaping landscapes and ecosystems, thus threatening biodiversity worldwide (Concepción et al. 2015; Theodorou 2022). As cities grow in size, wildlife habitats are increasingly fragmented or lost (Ancillotto et al. 2024, 2025; Dondina et al. 2025). Despite this, several species, including birds, small mammals, and invertebrates, adapt to urban environments (Santini et al. 2019; Fenoglio et al. 2021; Neate-Clegg et al. 2023; Dondina et al. 2025; Ancillotto et al. 2025). In this context, understanding how the public perceives and values urban wildlife is pivotal, as measuring public appreciation of urban biodiversity informs urban planning and conservation strategies (Kellert 1993; Manfredo et al. 2003, 2020), which may in turn promote a fair coexistence between humans and wildlife in densely populated areas (Serangeli et al. 2012; Cerri et al. 2020; Viviano et al. 2024; Peichar et al. 2025).

Appreciation of urban wildlife contributes directly to the success of biodiversity conservation policies and implementation actions (Clergeau et al. 2001; Soulsbury and White 2015). In this context, public support is paramount to successfully implement wildlife-friendly initiatives, such as the creation of green corridors, green roofs, adjustment of grass-cutting plans, plantation of native species, and regulation of free-ranging domestic pets (Kirkpatrick et al. 2012; Satoshi et al. 2014; Bassett et al. 2020; Teixera et al. 2022; Biella et al. 2025). Moreover, improving the relationships between human population and wildlife in urban areas can increase mental well-being, environmental awareness, and a deeper sense of connection with nature (Wolsko and Lindberg 2013; Sandifer et al. 2015).

However, assessing the full spectrum of public attitudes toward wildlife is challenging (Gargioni et al. 2021). Most studies in urban settings rely heavily on data from birds, as these species are often more visible, easily recognised, and associated with positive aesthetic or cultural values (Clergeau et al. 2001; Luna et al. 2019; Mori et al. 2020). Conversely, groups like mammals are less frequently studied in this regard, because urban mammals are often nocturnal, elusive, or less colourful, making them less immediately appealing or recognisable to the average observer (Moesch et al. 2024a, b).

Traditional surveys, while useful, face significant challenges in urban contexts, e.g. the limited response rate from citizens (Chen and Jim 2010; Gargioni et al. 2021). Time constraints, lack of interest, or survey fatigue often result in poor engagement (Verba et al. 1993; Ghafourifard 2024). Thus, there is an increasing need for creative, efficient, and appealing survey formats (Luna et al. 2019). Short questionnaires that rely on visual *stimuli*, especially those incorporating coloured plates of species, are proving more effective (Luna et al. 2019; Mori et al. 2020). These visuals not only reduce the load on respondents but also allow researchers to gather instinctive aesthetic or emotional reactions to different species (Luna et al. 2019; Mori et al. 2020). Such visual-based surveys have been used to studying avian preferences, given that many birds are visually distinctive and colour plays a strong role in human appreciation (Luna et al. 2019; Mori et al. 2020). In general, the most appreciated bird species in cities are those showing bright plumages or providing remarkable ecosystem services, such as pollination, seed dispersal, or pest control, as well as those that are not associated with harmful legends or negative stereotypes (Belaire et al. 2015; White et al. 2018; Andrade et al. 2022). Conversely, this approach reveals a gap when applied to mammals, where bright colouration is rare and aesthetic appeal is harder to quantify. However, using visual plates may be a faster and more intuitive way to capture preferences than direct, text-based questions, also for wild mammals.

Urban mammals like hedgehogs, bats, squirrels, or foxes may evoke strong reactions, based on folklore, perceived ecosystem services (e.g., pest control), or negative associations (e.g., disease, aggression, legends) (Bjerke and Østdahl 2004; Cerri et al. 2022; Viviano et al. 2025). For example, bats, despite being ecologically beneficial as insectivorous predators, often suffer from negative perceptions rooted in myths and legends or biased framing by media (Cerri et al. 2022). As to other wild mammals, data are lacking or still limited to few species (e.g., invasive coypus *Myocastor coypus*: Viviano et al. 2025). Species like urban Western European hedgehogs *Erinaceus europaeus*, which have a generally favourable public image, are likely to be well-regarded (Ribeiro et al. 2023). Conversely, species such as rats or bats, despite their ecological roles, may continue to suffer from low appreciation due to persistent myths and health concerns (Cerri et al. 2022; Aivelo 2023). Given the limits of existing data, and methodological constraints, researchers are beginning to model public preference based on known ecological and cultural variables (see Peichar et al. 2025 for birds).

The aim of this study was to examine and quantify potential differences in the way urban mammals are perceived in urban areas in ten cities in Italy.

Specifically, we aimed to assess how wild mammals are viewed and evaluated within urban environments, highlighting possible variations in public attitudes, opinions, and levels of appreciation (i.e., liked/disliked) toward them according to either respondent demographic traits or species characteristics. We predicted that (i) nocturnal species will be rated lower in appreciation than diurnal species (Basak et al. 2022; Sweet et al. 2023; Moesch et al. 2025); (ii) species associated with human-wildlife conflict will receive lower appreciation than species without conflict associations; (iii) familiarity with a species will positively predict appreciation scores.

## Materials and methods

### Field survey

**Fig. 1:**
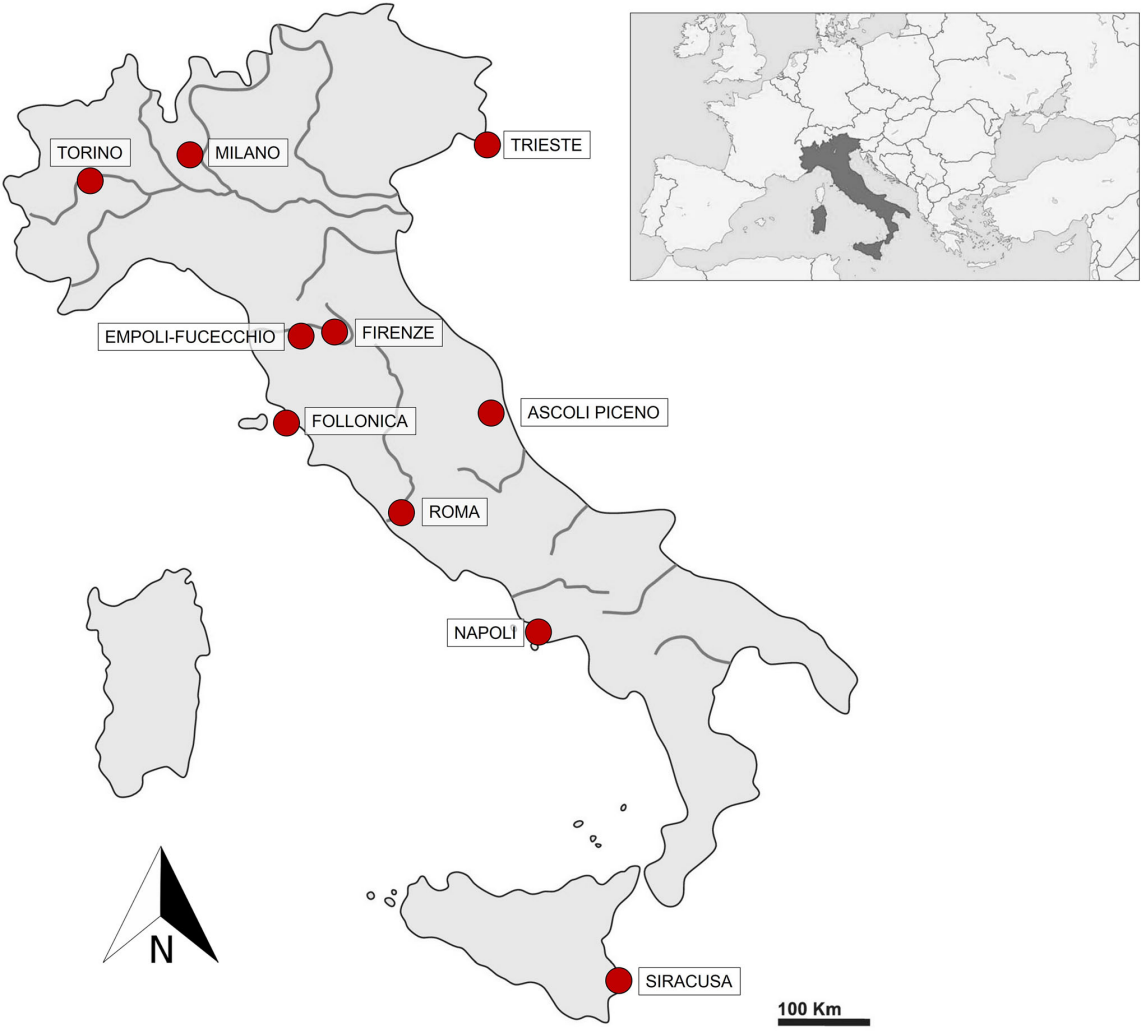
Location of the ten surveyed cities in Italy (N = 1000 total respondents): Torino, Milano, Trieste, Firenze, Empoli-Fucecchio, Follonica, Ascoli Piceno, Roma, Napoli and Siracusa

We used an in-person survey in 3–10 urban parks per city to a total of 1000 responses across 10 Italian cities during the period January–May 2025 (Fig. 1). The choice of 1000 participants was based on practical feasibility and on ensuring a sufficiently large and diverse sample to allow for meaningful comparisons across groups (i.e., gender, city). We selected cities representing a latitudinal (north–south) gradient across Italy and encompassing a wide range of population sizes, from 20,172 to 2,874,605 residents in 2024 (ISTAT 2025; Table 1), to capture variability in urban ecological contexts and public perception across different urban scales and geographic areas. Different authors conducted surveys in their respective cities to ensure that interviewers were familiar with the area and the surveyed population, and to reduce the likelihood of refusals. Surveys were conducted during all the days of the week in the afternoon, to maximise encounter rates. Each respondent was engaged for a maximum of five minutes. All participants were adults (i.e., over 18 years old), fully informed about the aims of the research, and freely completed the species ranking independently, to limit any influence by the researchers. All participants signed the consent form prior to the engagement with the researcher.

**Table 1:** Occurrence of different mammalian species in surveyed cities (Loy et al. 2025), which represent an index of species familiarity in each city

City	Number of inhabitants (2024)	Species														
		Wild boar	Crested porcupine	Hazel dormouse	Wolf	Rabbit	Marten	Pipistrelle	Hedgehog	Rat	Fox	Badger	Roe deer	Golden jackal	Squirrel	Coypu
Trieste	203 931	x		x	x	x	x	x	x	x	x	x	x	x	x	
Milano	1 366 453			x	x	x	x	x	x	x	x	x			x	x
Torino	1 710 521	x	x	x		x	x	x	x	x	x	x	x	x	x	x
Firenze	362 432	x	x	x	x	x	x	x	x	x	x	x	x	x	x	x
Empoli-Fucecchio	72 279	x	x	x	x	x	x	x	x	x	x	x	x	x	x	x
Follonica	20 172	x	x	x	x	x	x	x	x	x	x	x	x		x	x
Roma	2 874 605	x	x	x	x	x	x	x	x	x	x	x	x		x	x
Ascoli Piceno	45 312	x	x			x	x	x	x	x	x	x	x	x	x	x
Napoli	907 573					x	x	x	x	x	x					
Siracusa	116 247		x			x	x	x	x	x	x					

To increase the response rate and furtherly ensure participant engagement, we designed full-colour panels (hereafter defined as “plates”), based on the model proposed by Luna et al. (2019). Each plate was composed by photos of 15 selected mammal species known to thrive in urban areas across Italy, based on the most recent national mammal distribution assessment and specific studies focused on urban environments (Ancillotto et al. 2024, 2025; Loy et al. 2025). The selected species are those commonly or potentially present in major Italian cities (Tables 1, 2): the wild boar (*Sus scrofa*), the roe deer (*Capreolus capreolus*), the grey wolf (*Canis lupus*: hereafter, wolf), the red fox (*Vulpes vulpes*: hereafter, fox), the golden jackal (*Canis aureus*), the stone marten (*Martes foina*: hereafter, marten), the European badger (*Meles meles*: hereafter, badger), the hedgehog (*Erinaceus europaeus*), the Kuhl’s pipistrelle (*Pipistrellus kuhlii*: hereafter, bat), the black rat (*Rattus rattus*: hereafter, rat), the coypu (*Myocastor coypus*), the crested porcupine (*Hystrix cristata*), the red squirrel (*Sciurus vulgaris*: hereafter, squirrel), the hazel dormouse (*Muscardinus avellanarius*) and the rabbit (*Oryctolagus cuniculus*: Fig. S1 in Supplementary Material 1). The attitudes elicited by the stone marten should be considered equally applicable to the pine marten (*Martes martes*), a morphologically and ecologically similar species which can only be reliably distinguished by diagnostic traits even by trained specialists. Accordingly, the perception of Kuhl’s pipistrelle should be extended to bats as a whole, that of the red squirrel to the invasive grey squirrel (*Sciurus carolinensis*) in Turin and Milan (and possibly of the invasive Siberian chipmunk (*Eutamias sibiricus*) in Rome), that of the black rat to Norway rat (*R. norvegicus*), and that of the rabbit to the order Lagomorpha (e.g., *Lepus europaeus* and, for Milan and Turin, the invasive *Sylvilagus floridanus*). The plate included neutral high-quality photos of all species without background, without the intention of eliciting either positive or negative emotional responses per se, e.g. avoiding depictions such as predators with gaping jaws (see Luna et al. 2019). All images of the species were presented in a lateral orientation, facing left, to ensure greater neutrality of the plate. The same plate displaying all species was shown to every participant in all cities, regardless of the local presence or absence of each species.

**Table 2:** Traits of selected species (data from Boitani et al. 2003; Amori et al. 2008; Loy et al. 2025). “Cathemeral” refers to an activity pattern in animals in which activity occurs both during the day and night, without a strict preference for either. ^a^The pine marten has mostly a diurnal behaviour in natural environments. ^b^Grey squirrels occurring in Milano and Torino, and Siberian chipmunks occurring in some urban parks in Roma are introduced and invasive species

Species	Activity	Positive folklore	Human-wildlife conflict and negative folklore	Diet	Native/introduced
Wild boar	Nocturnal		x	Omnivorous	Native
Crested porcupine	Nocturnal	x	x	Herbivorous	Introduced
Hazel dormouse	Nocturnal			Herbivorous	Native
Wolf	Cathemeral		x	Carnivorous	Native
Rabbit	Diurnal	x		Herbivorous	Introduced
Marten	Nocturnal^a^		x	Carnivorous	Native
Bat	Nocturnal		x	Carnivorous	Native
Hedgehog	Nocturnal	x		Omnivorous	Native
Rat	Nocturnal		x	Omnivorous	Introduced
Fox	Nocturnal	x	x	Omnivorous	Native
Badger	Nocturnal			Omnivorous	Native
Roe deer	Cathemeral	x	x	Herbivorous	Native
Golden jackal	Cathemeral		x	Carnivorous	Native
Squirrel	Diurnal	x		Omnivorous	Native^b^
Coypu	Diurnal		x	Herbivorous	Introduced

In the first part of the survey, resident respondents were shown the plate on a Tablet, with images of the 15 mammal species (Fig. S1 in Supplementary Material 1). They were then asked to rank the species in order of preference based on which animals they would most like to see in the urban environment in which the survey was conducted. Higher-ranking species were interpreted as being perceived more positively by the public. More in general, species ranking between 1 and 5 were classified as “liked”, between 5 and 7 as “neutral/liked”, between 8 and 10 as “neutral/unliked” and between 11 and 16 as “unliked”, following Giuntini (2018). Public preferences were assessed using the average preference score assigned to each species and the frequency with which species were ranked among respondents’ top three choices. To streamline the survey process and reduce participant attrition, we collected only the gender of respondents, allowing for a balanced sample in terms of male and female representation (see Supplementary Material 1). We did not collect additional demographic data such as occupation or education level, as the primary aim of the study was to evaluate public perception of urban mammals based on a sample of 100 individuals per city. Participants were approached using convenience sampling in public parks and gardens across the study area. While individuals were selected on a voluntary basis rather than through a true random sampling, interviewers made a conscious effort to include equal numbers of men and women to ensure gender balance in the sample. Surveys were conducted with local residents in the urban areas under study to engage individuals who regularly interact with the urban environment. For the purposes of this study, a local resident was defined as a person living within the metropolitan area where the survey was conducted. Respondents who indicated that they were not residents were excluded from the survey.

### Statistical analyses

We compiled an Excel dataset containing all responses, organized by city and classified by gender; each row corresponded to a single anonymous respondent. Before running the analyses, the dataset was checked for completeness and consistency. Missing values were detected only in the *gender* variable, as some respondents had not provided this information. These cases were excluded from the gender-based analyses to ensure valid group contrasts but were retained in all other analyses.

To analyze public perceptions of urban mammals, a quantitative analysis was conducted using R version 4.2.2 (R Development Core Team 2023). The aim was to investigate citizen preferences toward different animal species, while also considering socio-demographic variables such as respondent gender and city of residence.

In the initial phase, two indicators were calculated for each species: the average preference score (on a scale from 1 = most appreciated to 15 = least appreciated) and the frequency with which each species was ranked among the top three preferences. In other words, participants were asked to rank all 15 species, and from these rankings we extracted the species placed in positions 1, 2, and 3 for the analyses. These indicators provided a synthetic overview of the most and least appreciated species across the sample. Variability was explored through boxplots of score distributions and hierarchical clustering of mean perception scores.

Gender differences were tested using the Wilcoxon rank-sum test with Bonferroni correction, as the data did not meet the assumption of normality (Shapiro–Wilk test, *p* < 0.001) (Divine et al. 2013). Geographic variation was assessed using the Kruskal–Wallis test, followed by Dunn’s post-hoc pairwise comparisons with Bonferroni correction (Graham et al. 2011).

Finally, to test whether the actual occurrence of each species influenced perceptions, cities were classified as presence or absence localities based on Loy et al. (2025). Differences in mean scores between these two conditions were assessed using Welch’s independent-samples t-test. This allowed for the identification of possible familiarity effects (linked to presence in a certain area and nocturnality/diurnality: Table 2) or, conversely, the influence of experiential distance (and potential human-wildlife conflicts) on attitudes toward urban wildlife (Boitani et al. 2003; Amori et al. 2008).

## Results

We obtained 100 full surveys in each city (N_tot_ = 1000), well gender-balanced (females, 49.2%; males 50.8%). Our study revealed significant variation in public perceptions of urban mammal species across Italian surveyed cities, influenced by respondent gender, geographical location, as well as by species traits and local occurrence within urban environments.

### Species-level preferences

**Fig. 2:**
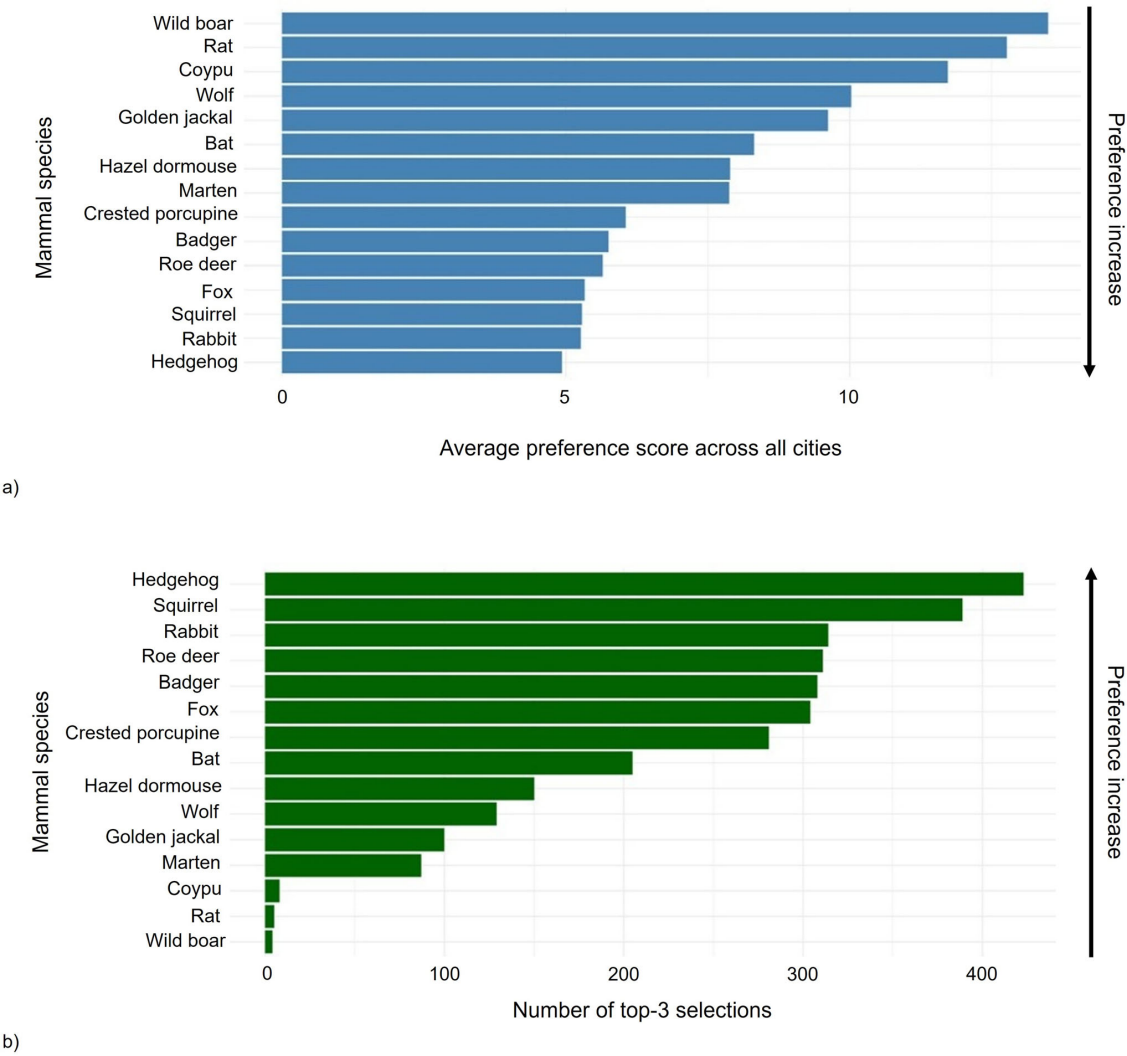
**a** Average preference (with standard error) scores assigned to each species in all cities (N = 1000; 1 = most preferred, 15 = least preferred); **b** top-3 preference frequencies for each mammal in all cities. Arrows highlight preference increases. In all cities, hedgehog showed the highest appreciation score, wild boar the lowest

The average appreciation scores assigned to each species are summarised in Fig. 2a, whereas the frequency with which species were ranked among respondent top three choices are shown in Fig. 2b. Hedgehog, rabbit, squirrel, and fox received the lowest average scores, indicating high preference, whereas coypu, rat, and wild boar obtained the highest scores, reflecting the overall lowest appreciation. These patterns were consistent across both indicators. Hedgehog emerged as the most frequently selected top-three species, followed by squirrel, rabbit, and roe deer, with similar frequencies. In contrast, wild boar, rat, and coypu were rarely included in top-three appreciation rankings.

**Fig. 3:**
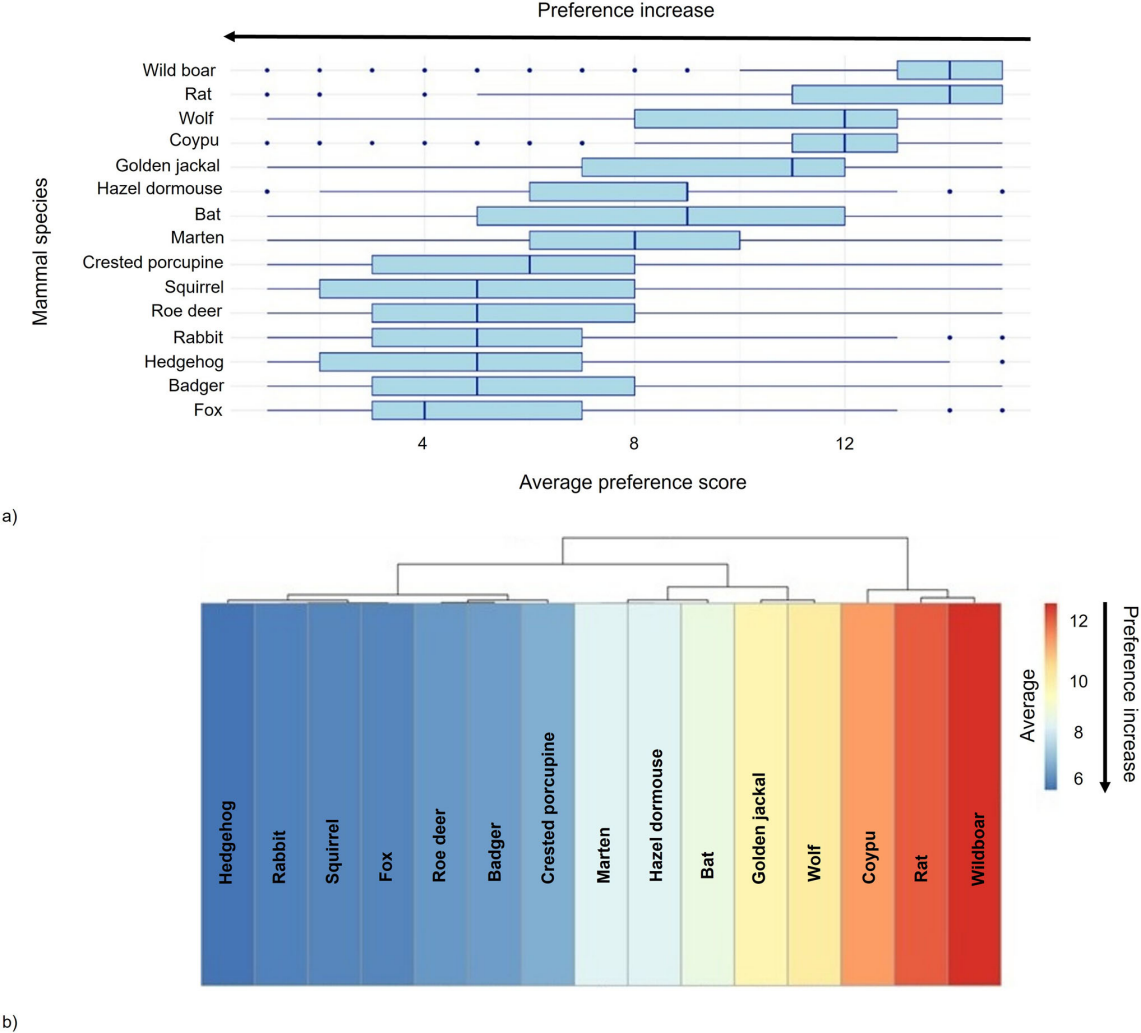
**a** Boxplots of public perception scores across species in different cities. Higher values indicate lower preference; **b** hierarchical clustering of mammals based on average perception scores. The dendrogram shows how different mammal species are grouped by their average social appreciation scores, where the height of the grey branches indicates the level of similarity between them, with shorter branches connecting species that are more alike. Arrows highlight preference increases

The grey wolf showed evidence of polarised perceptions. Despite receiving relatively low appreciation scores, the wolf was frequently ranked among top-three preferences, suggesting heterogeneity in public attitudes. This polarization was further supported by the distribution of scores (Fig. 3a).

Finally, hierarchical cluster analysis (Fig. 3b) identified three main clusters based on perception profiles. Cluster 1 included the least appreciated species (coypu, rat, wild boar). Cluster 2 included favourably and neutrally evaluated species (hedgehog, fox, rabbit, squirrel, roe deer, badger, crested porcupine). Cluster 3 included species with more variable evaluations, including golden jackal, wolf, marten, and bat.

### Gender differences

**Fig. 4:**
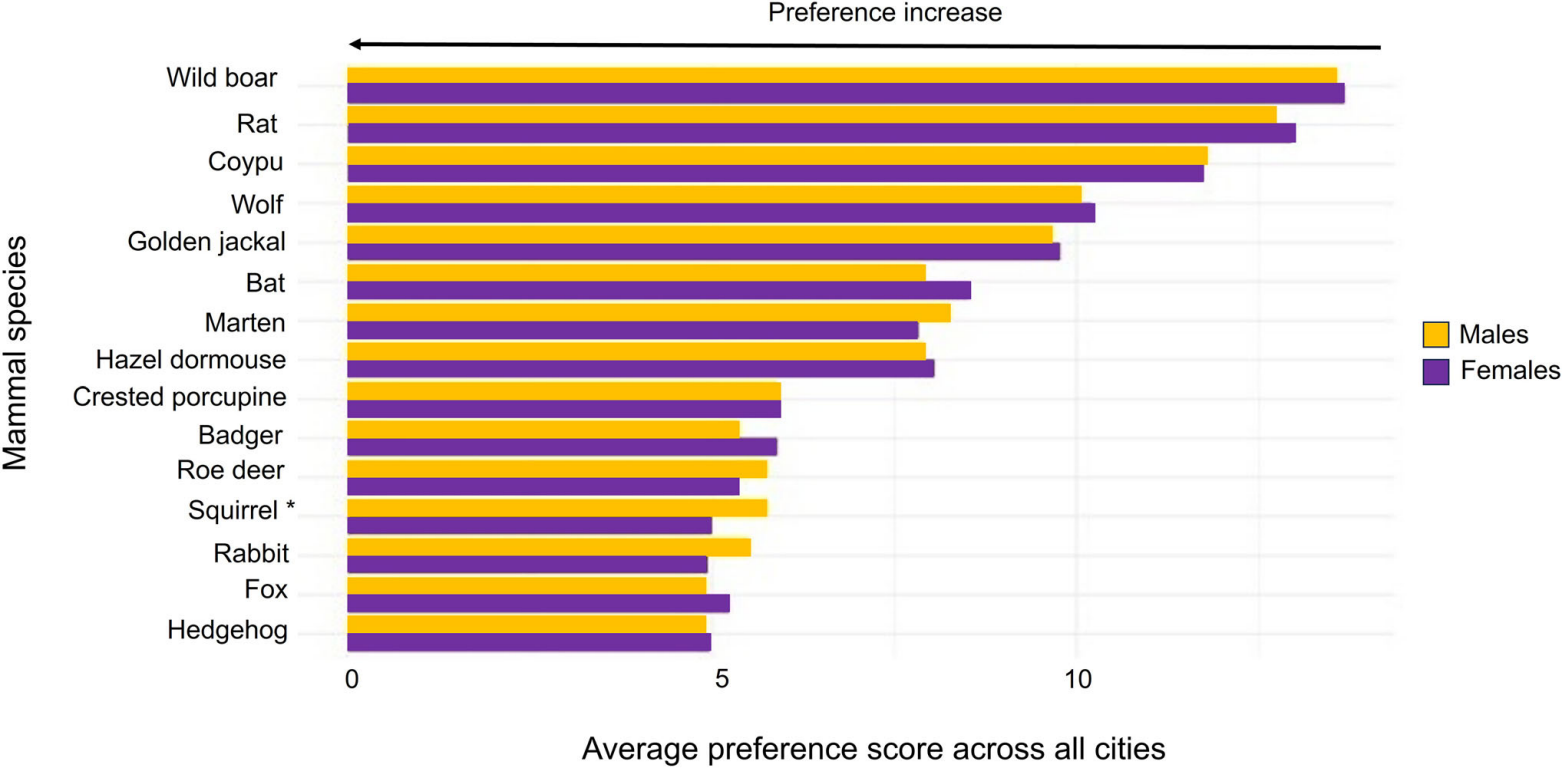
Average perception scores (with standard error) by mammal species and gender of respondents. Arrows highlight preference increases. *Significant difference (only for squirrels, more appreciated by females than by males). Bars represent mean scores for males (yellow) and females (purple). Higher scores indicate lower preference

Gender-based analysis was conducted on 901 surveys, as cases with missing values for the gender variable (NA) were excluded from this analysis. Results revealed a statistically significant difference in the perception of squirrels. Specifically, a Wilcoxon rank-sum test indicated that female respondents rated squirrels as significantly more preferred (i.e., with lower scores) than males (MdnM = 6, IQR = 3–8; MdnF = 4, IQR = 2–7), W = 114 520.5, nF = 492, nM = 409, unadjusted *p* < 0.001, Bonferroni-adjusted *p* = 0.0048, r = 0.12. Other gender-related trends, such as higher female scores for rabbit and roe deer or higher male scores for badger, did not reach statistical significance after Bonferroni correction (Fig. 4).

### Geographic variation

**Fig. 5:**
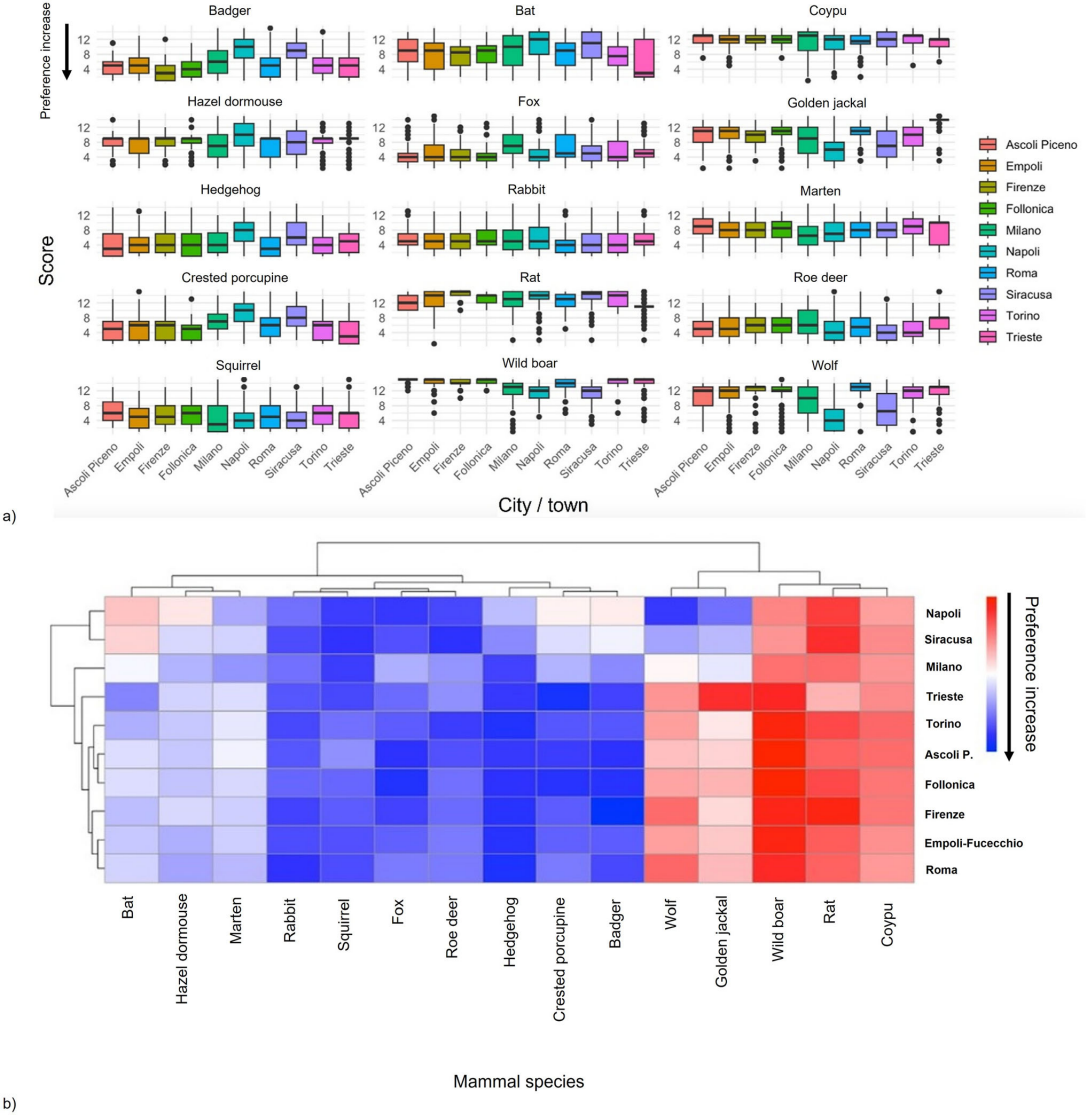
**a** Average perception scores by mammal species per city; **b** heatmap of public perception scores by species and city. Arrows highlight preference increases. The dendrograms on the top and left of the heatmap illustrate clustering relationships. They show how species (top) and cities (left) are grouped based on the similarity of their preference scores, revealing patterns in social appreciation. Differences among cities were confirmed by Kruskal–Wallis tests and Dunn’s post-hoc pairwise comparisons with Bonferroni correction (see Tables S1 and S2 in Supplementary Material 2)

Perception varied across cities (Fig. 5a). Kruskal–Wallis tests, followed by Dunn’s post-hoc pairwise comparisons with Bonferroni correction, confirmed the presence of multiple statistically significant differences among cities, highlighting distinct local patterns of appreciation toward different mammal species (Tables S1, S2, Supplementary Material 2).

As shown in Fig. 5b, several cities in central Italy, such as Roma, Empoli-Fucecchio, Firenze, Follonica, and Ascoli Piceno, exhibited broadly similar perceptual profiles of urban mammals. In contrast, other cities diverged markedly, particularly with respect to some species. For example, both the golden jackal and the wolf received significantly lower evaluations in Siracusa and Napoli compared to Firenze, Roma, and Empoli-Fucecchio (e.g., golden jackal: Firenze vs Siracusa, z = 3.34, Bonferroni *p* = 0.037; Napoli vs. Roma, z = − 7.90, Bonferroni *p* < 0.001; wolf: Napoli vs. Roma, z = − 11.82, Bonferroni *p* < 0.001; Roma—Siracusa, z = 8.26, Bonferroni *p* < 0.001; see Table S2 in Supplementary Material 2). Moreover, species that are generally more appreciated in other contexts (e.g., bats, hazel dormouse, badger, crested porcupine) were also perceived more negatively in Napoli and Siracusa than in other cities (e.g., badger: Firenze vs. Napoli, z = − 12.1, Bonferroni *p* < 0.001; bat: Firenze vs. Napoli, z = − 4.84, Bonferroni *p* < 0.001; dormouse: Napoli vs. Roma, z = 5.42, Bonferroni *p* < 0.001; porcupine: Follonica vs. Napoli, z = − 9.81, Bonferroni *p* < 0.001; see Table S2 in Supplementary Material 2).

At the opposite end of the country, Trieste stands out markedly from the other cities, particularly from Napoli and Siracusa, by showing more negative evaluations of several species (e.g., wild boar: Napoli vs. Trieste, z = − 8.92, Bonferroni *p* < 0.001; porcupine: Siracusa vs. Trieste, z = 8.46, Bonferroni *p* < 0.001; rat: Siracusa vs. Trieste, z = 9.37, Bonferroni *p* < 0.001; see Table S2 in Supplementary Material 2). Even species generally regarded negatively, such as the rat, were evaluated relatively less unfavourably in Trieste, while bats were perceived more positively compared to other Italian cities.

Finally, Milano occupies a moderate position, showing a distinct but moderate perceptual profile. For species such as the wolf and the golden jackal, Milano does not display the hostility observed in cities like Torino, Roma, Trieste, and Follonica, nor the relatively higher appreciation found in Siracusa and Napoli (e.g., golden jackal: Milano vs. Roma, z = − 3.41, Bonferroni *p* = 0.029; wolf: Milano vs. Roma, z = − 4.91, Bonferroni *p* < 0.001; see Table S2 in Supplementary Material 2).

### Species presence and familiarity

**Fig. 6:**
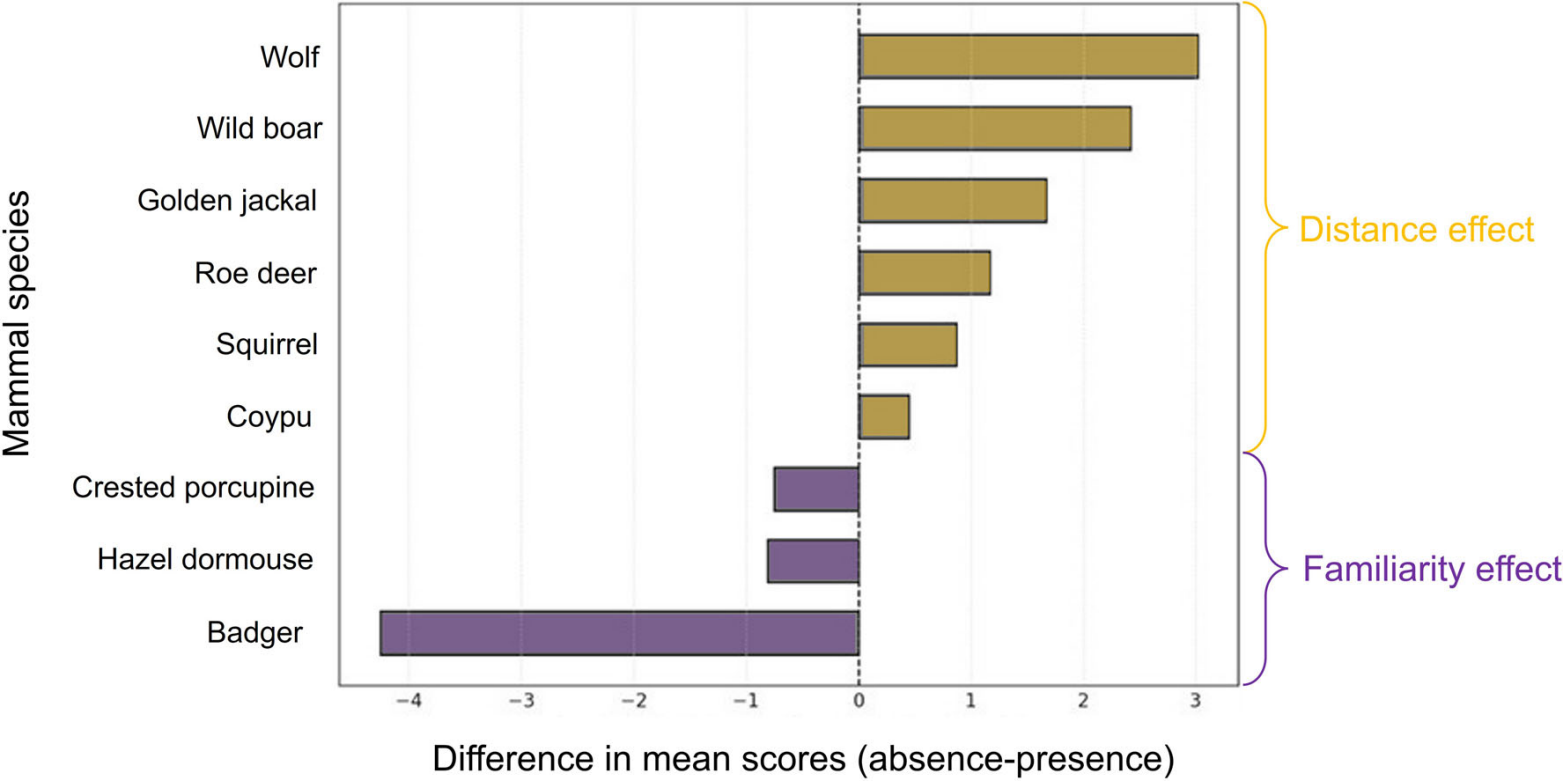
Mean difference between cities where the species is present and those where it is absent. The graph shows only species for which the differences are statistically significant. The x-axis represents the difference in average scores between cities where the species is present and those where it is absent. Badger, hazel dormouse, and crested porcupine were significantly more appreciated in cities where they occur (familiarity effect), whereas wolf and jackal were rated higher in cities where they were absent (distance effect)

The relationship between species perception and their local actual occurrence was also examined (Fig. 6). T-tests revealed contrasting patterns depending on the species (Table S3 in Supplementary Material 2). For instance, badger was evaluated more negatively in cities where the species was absent (Mabsent = 9.07, SD = 3.06) than in cities where it was present (Mpresent = 4.82, SD = 3.02; Welch’s t (318.3) = 17.38, *p* < 0.001, Cohen’s d = 1.40). A similar trend was observed for roe deer (Mabsent = 4.71, SD = 3.21; Mpresent = 5.87, SD = 3.05; t(308.7) = − 4.54, *p* < 0.001, d = − 0.37). Conversely, large mammals such as wolf, wild boar, and golden jackal were perceived more negatively (thus obtaining lower scores) in cities where they were present (Mabsent = 8.07 and 9.11, SD = 3.51 and 2.77; Mpresent = 6.42 and 5.13, SD = 3.90 and 4.01; t (413.2 and 388.8) = 3.58 and 3.33, *p* < 0.001, d = 0.28 and 0.30).

The hazel dormouse was rated higher, thus less appreciated, in cities where it was absent (Mabsent = 8.53, SD = 3.49; Mpresent = 7.69, SD = 2.96; t (518.4) = 3.58, *p* < 0.001, d = 0.26). Similarly, squirrels received higher scores in absence cities (Mabsent = 4.66, SD = 3.45; Mpresent = 5.49, SD = 3.31; t(311.0) = –3.02, *p* = 0.003, d = − 0.24). Conversely, coypus were poorly appreciated both in absence and in presence cities (Mabsent = 11.47, SD = 2.49; Mpresent = 11.90, SD = 2.14; t(525.1) = − 2.58, *p* = 0.010, d = − 0.19). Finally, crested porcupine was marginally less appreciated in absence cities (Mabsent = 6.57, SD = 4.13; Mpresent = 5.95, SD = 3.23; t(272.6) = 1.97, *p* = 0.050, d = 0.17).

## Discussion

Public perception can act as either a barrier or a catalyst for urban biodiversity conservation. Understanding how people perceive different mammal species is therefore essential, as attitudes can influence both management decisions and coexistence in urban environments. Our study provides insights into these perceptions, highlighting which species are viewed more positively or negatively and how factors such as familiarity, exposure, or cultural context shape these preferences. We surveyed how citizens perceive different species of urban-thriving mammals in 10 Italian cities, revealing a multifaceted spectrum of appreciation which varies by species, gender, geography, and people familiarity with species. While previous work has heavily focused on birds (Hedblom et al. 2014; Luna et al. 2019; Mori et al. 2020), we here fill an essential gap by assessing perceptions of urban mammal communities, many of which are nocturnal, elusive, or socially stigmatised. General aesthetic appeal related to our study species and perceived ecological utility seem to represent major drivers of public preference, particularly in urban ecosystems (see De Pinho et al. 2014; Drouilly et al. 2021 for ungulates and large carnivores, respectively). Hedgehog, squirrel, rabbit, and roe deer were consistently preferred, aligning with earlier studies highlighting positive public sentiment towards visible (diurnal or, in the case of roe deer and hedgehog, very widespread, thus fulfilling our prediction i), charismatic (as being parts of several cartoons, in particular for roe deer, squirrels and rabbits) fauna (Morris 1987). Hedgehogs also appear to benefit from a combination of perceived cuteness, ecological usefulness such as pest control, and cultural affection, making them effective representatives of urban wildlife appreciation (Ribeiro et al. 2023). In contrast, species like rats, wild boar, and coypu were persistently rated negatively, as usually being perceived as invasive and generating conflicts with human activities and wellness, in line with past research (Marshall and Murphy 2003; Zhou et al. 2023; Viviano et al. 2024), and our prediction ii. Marshall and Murphy (2003) and German and Latkin (2016) suggested that this pattern reflects deep-seated socio-cultural stigmas as well as concerns about disease transmission (particularly rats), aggression (i.e., large carnivores as wolves and jackals), or property damage (e.g., foxes and martens on poultries). Our geographical variability indicates that indeed, social-cultural factors may play a role in the appreciation of species. The low appreciation for coypus reflects literature data on perception towards this invasive species (Viviano et al. 2024). Past studies found polarised responses towards wolf, being simultaneously feared and admired (Behr et al. 2017; Albert et al. 2018; Prokop et al. 2024), a pattern reflected by our results. While some respondents ranked wolves among their top preferences, others scored them poorly, reflecting the broader European debates about large carnivores in human-dominated landscapes (Zorondo-Rodriguez et al. 2020; Franchini et al. 2025). Such polarisation most likely reflects the tension between symbolic admiration and real or perceived conflict risk (Prokop et al. 2024). This pattern may thus support a proximity-related conflict effect. Notably, both wolf and jackal were evaluated more positively in cities where they were absent (e.g., Napoli and Siracusa), suggesting a “distance effect,” whereby species not directly encountered may be viewed more idealistically.

In our study, women rated squirrels significantly higher than men, with other non-significant trends favouring also rabbit and roe deer. Previous research suggested that females are generally more empathetic toward non-threatening, visually appealing animals (Herzog 2007). This gender-based pattern may stem from evolutionary or sociocultural factors, which influence aesthetic preference or perceived threat levels (Herzog 2007). The observed gender difference for squirrels may reflect differences in exposure or familiarity, with women potentially having more frequent or positive encounters with this species. This indicates that sociocultural factors, such as daily experiences and interactions with certain animals, could underlie gender-specific preferences, rather than evolutionary explanations or perceived threat levels. However, most species showed no significant gender difference, indicating that if gender may shape preferences for select species, other factors including cultural exposure or urban experience may exert stronger influence.

Significant geographic variation in species perceptions underscores the context-dependence of human-wildlife relationships (cf. Kiley et al. 2017). Golden jackals and badgers, for instance, were viewed more positively in southern cities such as Siracusa and Napoli, suggesting that regional culture or differing wildlife exposure may modulate public attitudes. Conversely, hedgehogs and squirrels, though generally appreciated, received lower ratings in these same cities, possibly due to their local absence reduced visibility or local ecological narratives. Large carnivores (i.e., wolves and jackals) were rated more favourably in cities where they were not present, consistent with the notion that their absence fosters idealization while direct experience may fuel fear and other negative sentiments (Albert et al. 2018; Prokop et al. 2024). Such patterns caution against universalising conservation messaging and highlight the need for localised education efforts addressed to community experiences and species presence. The “familiarity effect” that species are more positively perceived where they are present was only partly supported (see our prediction iii). For instance, crested porcupines, hazel dormice, and badgers were more appreciated in cities where they occurred, indicating that repeated positive exposure can trigger positive sentiment (Mori et al. 2025). However, the reverse was true for wolves and wild boar, reinforcing that familiarity can also exacerbate negative views, especially when species are associated with human-wildlife conflicts (Zorondo-Rodriguez et al. 2020). These findings highlight a critical challenge for urban biodiversity planning, how to promote coexistence where ecological presence does not automatically translate into public support. Addressing these gaps may require coupling wildlife-friendly infrastructure with targeted communication strategies to dispel myths and emphasise potential ecosystem services (see Cerri et al. 2022).

The use of visually based low-cognitive-load surveys, i.e. not requiring detailed knowledge on the species, is known to prove highly effective in capturing instinctive aesthetic and emotional responses, allowing for engagement with diverse populations due to ease in administration (Luna et al. 2019). Extending this visual survey method to include behavioural or acoustic *stimuli*, particularly for less visible species, e.g. bats, may yield more nuanced insights into public biases and fears, and provide a more inclusive approach to respondent selection (e.g., persons with visual impairment).

Understanding public perceptions of urban wildlife is pivotal, as attitudes can directly influence conservation priorities and management decisions, with non-preferred species potentially facing neglect or removal pressures (e.g., La Morgia et al. 2017; Gargioni et al. 2021; Viviano et al. 2024). Our study highlights how preferences vary with factors such as species familiarity and proximity, suggesting that actively engaging citizens could help mitigate conflicts arising from negative perceptions. In this context, integrating citizen science into urban mammal monitoring may offer a promising approach: it not only fills gaps between experience, perception, and scientific knowledge, but also provides a baseline for future research, and informs targeted outreach and education in areas where similar species are common.

Despite using neutral images (Fig. S1 in Supplementary Material S1), we acknowledge the potential for sample bias, as participants may have differed in age, which could have in turn affected their responses (see Sweet and Weisser 2025). Moreover, we only measured the level of appreciation, and our ranking approach does not necessarily capture absolute like/dislike attitudes but rather relative preferences among species, and we cannot exclude participants may also have experienced difficulties in identifying some species correctly. Future studies could implement a mixed-method approach to explore further the mechanisms for acceptability patterns or include additional construct measures to increase explanatory power.

In general, we found that perceptions in our ten Italian cities were consistent with those observed in other European countries regarding the same species, with more charismatic species being preferred and less charismatic or invasive ones being less favored (e.g., UK: Baker et al. 2020; Poland: Basak et al. 2022; Germany: Sweet et al. 2024; Moesch et al. 2024c). However, changes in population density can alter public perceptions of a species, transforming initial admiration and curiosity into aversion when individuals become overly abundant, as shown by long-term studies in urban areas characterized by low species richness and high densities of a few wild mammal species (Basak et al. 2022). To our knowledge, this is the first comparative study within a single country that includes such a large number of cities spanning all latitudes from south to north.

To conclude, our study provides baseline data on public perceptions of urban wildlife, which may inform management decisions and communication strategies. By highlighting how attitudes vary across species and urban contexts, these findings can help managers identify areas or species where interventions, whether for coexistence, control, or mitigation, are most likely to align with public acceptability. Previous research has shown that outreach and education can influence perceptions of less preferred species (e.g., Van Dalum 2013; Thompson et al. 2016; Mwebaze et al. 2018), and our results contribute complementary insight into existing attitudes that such initiatives might build upon. Overall, integrating knowledge of public perceptions into management planning can support evidence-based, context-sensitive strategies for urban wildlife governance.

## Supplementary Information

Below is the link to the electronic supplementary material.

Supplementary file 1 (PDF 721 KB)

## Data Availability

Data used in this study are available in the Supplementary Material.

## References

[CR1] Aivelo, T. 2023. School students’ attitudes towards unloved biodiversity: Insights from a citizen science project about urban rats. *Environmental Education Research* 29: 81–98. 10.1080/13504622.2022.2140125.

[CR2] Albert, C., G. M. Luque, and F. Courchamp. 2018. The twenty most charismatic species. *PLoS ONE* 13: e0199149. 10.1371/journal.pone.0199149. 29985962 10.1371/journal.pone.0199149 PMC6037359

[CR3] Amori, G., L. Contoli, and A. Nappi. 2008. Fauna d’Italia, Mammalia II: Erinaceomorpha, Soricomorpha, Lagomorpha, Rodentia. Edizioni Calderini, Bologna, Italia.

[CR4] Ancillotto, L., G. Amori, D. Capizzi, B. Cignini, M. Zapparoli, and E. Mori. 2024. No city for wetland species: Habitat associations affect mammal persistence in urban areas. *Proceedings of the Royal Society B* 291: 20240079. 10.1098/rspb.2024.0079. 38471547 10.1098/rspb.2024.0079 PMC10932704

[CR5] Ancillotto, L., G. Guerri, P. Agnelli, L. Bonora, M. Maggioni, M. Morabito, and E. Mori. 2025. Past present: Extinction debt of forest mammals from urban areas. *Biological Conservation* 306: 111143. 10.1016/j.biocon.2025.111143.

[CR6] Andrade, R., K. L. Larson, J. Franklin, S. B. Lerman, H. L. Bateman, and P. S. Warren. 2022. Species traits explain public perceptions of human–bird interactions. *Ecological Applications* 32: e2676. 10.1002/eap.2676. 35582734 10.1002/eap.2676

[CR7] Baker, S. E., S. A. Maw, P. J. Johnson, and D. W. Macdonald. 2020. Not in my backyard: Public perceptions of wildlife and “pest control” in and around UK homes, and Local Authority “pest control.” *Animals* 10: 222. 10.3390/ani10020222. 32019151 10.3390/ani10020222 PMC7071040

[CR8] Basak, S. M., M. S. Hossain, D. T. O’Mahony, H. Okarma, E. Widera, and I. A. Wierzbowska. 2022. Public perceptions and attitudes toward urban wildlife encounters: A decade of change. *Science of the Total Environment* 834: 155603. 10.1016/j.scitotenv.2022.155603. 35523348 10.1016/j.scitotenv.2022.155603

[CR9] Bassett, I. E., E. J. McNaughton, G. D. Plank, and M. C. Stanley. 2020. Cat ownership and proximity to significant ecological areas influence attitudes towards cat impacts and management practices. *Environmental Management* 67: 30–41. 10.1007/s00267-020-01289-2. 10.1007/s00267-020-01289-2 32318754

[CR10] Behr, D. M., A. Ozgul, and G. Cozzi. 2017. Combining human acceptance and habitat suitability in a unified socio-ecological suitability model: A case study of the wolf in Switzerland. *Journal of Applied Ecology* 54: 1919–1929. 10.1111/1365-2664.12880.

[CR11] Belaire, J. A., L. M. Westphal, C. J. Whelan, and E. S. Minor. 2015. Urban residents’ perceptions of birds in the neighborhood: Biodiversity, cultural ecosystem services, and disservices. *The Condor* 117: 192–202. 10.1650/CONDOR-14-128.1.

[CR12] Biella, P., L. Bani, E. Caprio, F. Cochis, O. Dondina, V. Fiorilli, A. Genre, R. Gentili, et al. 2025. Biodiversity-friendly practices to support urban nature across ecosystem levels in green areas at different scales. *Urban Forestry & Urban Greening* 105: 128682. 10.1016/j.ufug.2025.128682.

[CR13] Bjerke, T., and T. Østdahl. 2004. Animal-related attitudes and activities in an urban population. *Anthrozoös* 17: 109–129. 10.2752/089279304786991783.

[CR14] Boitani, L., S. Lovari, and A. Vigna Taglianti. 2003. Fauna dItalia. Mammalia III. Carnivora-Artiodactyla. Edagricole Calderini Il Sole 24ore, Bologna, Italy.

[CR15] Cerri, J., E. Mori, R. Zozzoli, A. Gigliotti, A. Chirco, and S. Bertolino. 2020. Managing invasive Siberian chipmunks *Eutamias sibiricus* in Italy: A matter of attitudes and risk of dispersal. *Biological Invasions* 22: 603–616. 10.1007/s10530-019-02115-5.

[CR16] Cerri, J., E. Mori, L. Ancillotto, D. Russo, and S. Bertolino. 2022. COVID-19, media coverage of bats and related Web searches: A turning point for bat conservation? *Mammal Review* 52: 16–25. 10.1111/mam.12261. 34548738 10.1111/mam.12261 PMC8447447

[CR17] Chen, W. Y., and C. Y. Jim. 2010. Resident motivations and willingness-to-pay for urban biodiversity conservation in Guangzhou (China). *Environmental Management* 45: 1052–1064. 10.1007/s00267-010-9478-2. 20354852 10.1007/s00267-010-9478-2

[CR18] Clergeau, P., G. Mennechez, A. Sauvage, and A. Lemoine. 2001. Human perception and appreciation of birds: A motivation for wildlife conservation in urban environments of France. In *Avian ecology and conservation in an urbanizing world*, ed. J. M. Marzluff, R. Bowman, and R. Donnelly, 69–88. London: Springer.

[CR19] Concepción, E. D., M. Moretti, F. Altermatt, M. P. Nobis, and M. K. Obrist. 2015. Impacts of urbanisation on biodiversity: The role of species mobility, degree of specialisation and spatial scale. *Oikos* 124: 1571–1582. 10.1111/oik.02166.

[CR20] De Pinho, J. R., C. Grilo, R. B. Boone, K. A. Galvin, and J. G. Snodgrass. 2014. Influence of aesthetic appreciation of wildlife species on attitudes towards their conservation in Kenyan agropastoralist communities. *PLoS ONE* 9: e88842. 10.1371/journal.pone.0088842. 24551176 10.1371/journal.pone.0088842 PMC3925186

[CR21] Divine, G., H. J. Norton, R. Hunt, and J. Dienemann. 2013. A review of analysis and sample size calculation considerations for Wilcoxon tests. *Anesthesia & Analgesia* 117: 699–710. 10.1213/ANE.0b013e31827f53d7. 23456667 10.1213/ANE.0b013e31827f53d7

[CR22] Dondina, O., P. Tirozzi, A. Viviano, E. Mori, V. Orioli, N. Tommasi, A. Tanzi, L. Bazzoli, et al. 2025. Spatial and habitat determinants of small-mammal biodiversity in urban green areas: Lessons for nature-based solutions. *Urban Forestry & Urban Greening* 104: 128641. 10.1016/j.ufug.2024.128641.

[CR23] Drouilly, M., N. Nattrass, and M. J. O’Riain. 2021. Beauty or beast? Farmers’ dualistic views and the influence of aesthetic appreciation on tolerance towards black-backed jackal and caracal. *PLoS ONE* 16: e0248977. 10.1371/journal.pone.0248977. 33740027 10.1371/journal.pone.0248977 PMC7978353

[CR24] Fenoglio, M. S., A. Calviño, E. González, A. Salvo, and M. Videla. 2021. Urbanisation drivers and underlying mechanisms of terrestrial insect diversity loss in cities. *Ecological Entomology* 46: 757–771. 10.1111/een.1304.

[CR25] Franchini, M., A. Švajda, M. Uhrín, and P. Prokop. 2025. People and bears: Evaluating public attitudes to foster human–carnivore coexistence in Slovakia. *Hystrix the Italian Journal of Mammalogy*. 10.4404/hystrix-00774-2025.

[CR26] Gargioni, C., A. Monaco, G.F. Ficetola, L. Lazzeri, and E. Mori. 2021. From the Andes to the Apennines: Rise and fall of a free-ranging population of feral llamas. *Animals* 11: 857. 10.3390/ani11030857. 33803497 10.3390/ani11030857 PMC8003056

[CR27] German, D., and C. A. Latkin. 2016. Exposure to urban rats as a community stressor among low-income urban residents. *Journal of Community Psychology* 44: 249–262. 10.1002/jcop.21762.

[CR28] Ghafourifard, M. 2024. Survey fatigue in questionnaire based research: The issues and solutions. *Journal of Caring Sciences* 13: 148–149. 10.34172/jcs.33287. 39974826 10.34172/jcs.33287 PMC11833437

[CR29] Giuntini, S. 2018. Alieni tra noi: percezione pubblica e tolleranza sociale verso i parrocchetti dal collare invasivi in Italia. Bachelor Dissertation at the University of Florence, Scienze Faunistiche, Florence, Italy.

[CR30] Graham, M. A., S. Chakraborti, and S. W. Human. 2011. A nonparametric exponentially weighted moving average signed-rank chart for monitoring location. *Computational Statistics and Data Analysis* 55: 2490–2503. 10.1016/j.csda.2011.02.013.

[CR100] Herzog, H.A. 2007. Gender differences in human–animal interactions: A review. *Anthrozoös* 20: 7–21.

[CR31] Hedblom, M., E. Heyman, H. Antonsson, and B. Gunnarsson. 2014. Bird song diversity influences young people’s appreciation of urban landscapes. *Urban Forestry & Urban Greening* 13: 469–474. 10.1016/j.ufug.2014.04.002.

[CR32] ISTAT. 2025. May 2025, Number of inhabitants in Italian cities, from https://www.istat.it. Accessed on 15 May 2025

[CR33] Kellert, S. R. 1993. Attitudes, knowledge, and behavior toward wildlife among the industrial superpowers: United States, Japan, and Germany. *Journal of Social Issues* 49: 53–69. 10.1111/j.1540-4560.1993.tb00908.x.

[CR34] Kiley, H. M., G. B. Ainsworth, W. F. van Dongen, and M. A. Weston. 2017. Variation in public perceptions and attitudes towards terrestrial ecosystems. *Science of the Total Environment* 590: 440–451. 10.1016/j.scitotenv.2016.12.179. 28291613 10.1016/j.scitotenv.2016.12.179

[CR35] Kirkpatrick, J. B., A. Davison, and G. D. Daniels. 2012. Resident attitudes towards trees influence the planting and removal of different types of trees in eastern Australian cities. *Landscape and Urban Planning* 107: 147–158. 10.1016/j.landurbplan.2012.05.015.

[CR36] La Morgia, V., D. Paoloni, and P. Genovesi. 2017. Eradicating the grey squirrel *Sciurus carolinensis* from urban areas: An innovative decision-making approach based on lessons learnt in Italy. *Pest Management Science* 73:354–363. 10.1002/ps.4352. 27367228 10.1002/ps.4352

[CR37] Loy, A., M. Bon, M. Di Febbraro, D. Baisero, and G. Amori. 2025. Atlas of Mammals in Italy. Edizioni Belvedere, Latina, Italy.

[CR38] Luna, A., P. Edelaar, and A. Shwartz. 2019. Assessment of social perception of an invasive parakeet using a novel visual survey method. *Neobiota* 46: 71–89. 10.3897/neobiota.42.31017.

[CR39] Manfredo, M., T. Teel, and A. Bright. 2003. Why are public values toward wildlife changing? *Human Dimensions of Wildlife* 8: 287–306. 10.1080/716100425.

[CR40] Manfredo, M. J., E. G. Urquiza-Haas, A. W. D. Carlos, J. T. Bruskotter, and A. M. Dietsch. 2020. How anthropomorphism is changing the social context of modern wildlife conservation. *Biological Conservation* 241: 108297. 10.1016/j.biocon.2019.108297.

[CR41] Marshall, P. A., and R. G. Murphy. 2003. Investigating residents’ perceptions of urban rodents in Manchester, UK. *ACIAR Monograph Series* 96: 473–476.

[CR42] Moesch, S. S., T. Wellmann, D. Haase, and M. Bhardwaj. 2024a. Mammal Mia: A review on how ecological and human dimension research on urban wild mammals can benefit future biophilic cities. *Basic and Applied Ecology* 79: 90–101. 10.1016/j.baae.2024.05.004.

[CR43] Moesch, S. S., T. M. Straka, J. M. Jeschke, D. Haase, and S. Kramer-Schadt. 2024b. The good, the bad, and the unseen: Wild mammal encounters influence wildlife preferences of residents across socio-demographic gradients. *Ecology & Society* 29: 6. 10.5751/ES-15211-290306.

[CR44] Moesch, S. S., Z. Ladds, and R. A. Francis. 2024c. Life in the deadlands: unearthing reasons for visiting and visitor perceptions of wildlife in London’s Magnificent Seven cemeteries. *Journal of Urban Ecology* 10: juae022. 10.1093/jue/juae022.

[CR45] Moesch, S., M. Sultana, G. Peerenboom, and I. Storch. 2025. Nocturnal neighbors: Exploring residents’ perceptions of urban wildlife related to animal traits identified by camera traps and literature. *Authorea*. 10.22541/au.174350190.06779062/v1.

[CR46] Mori, E., G. Onorati, and S. Giuntini. 2020. Loud callings limit human tolerance towards invasive parakeets in urban areas. *Urban Ecosystems* 23: 755–760. 10.1007/s11252-020-00954-y.

[CR47] Mori, E., A. Viviano, L. Ancillotto, G. Onorati, and C. Tattoni. 2025. Press coverage and public perception of crested porcupines in urban and rural areas of Italy. *J Nat Cons* 84: 126786. 10.1016/j.jnc.2024.126786.

[CR48] Morris, P. A. 1987. Changing attitudes towards British mammals. *Biological Journal of the Linnean Society* 32: 225–233. 10.1111/j.1095-8312.1987.tb00429.x.

[CR49] Mwebaze, P., G. C. Marris, M. Brown, A. MacLeod, G. Jones, and G. E. Budge. 2018. Measuring public perception and preferences for ecosystem services: A case study of bee pollination in the UK. *Land Use Policy* 71: 355–362. 10.1016/j.landusepol.2017.11.045.

[CR50] Neate-Clegg, M. H., B. A. Tonelli, C. Youngflesh, J. X. Wu, G. A. Montgomery, C. H. Şekercioğlu, and M. W. Tingley. 2023. Traits shaping urban tolerance in birds differ around the world. *Current Biology* 33: 1677–1688. 10.1016/j.cub.2023.03.024. 37023752 10.1016/j.cub.2023.03.024

[CR51] Peichar, L., C. C. Rega-Brodsky, L. B. Vazquez, and I. MacGregor-Fors. 2025. Bird-mediated ecosystem services and disservices in cities and towns. *Frontiers in Ecology and the Environment* 2025: e2835. 10.1002/fee.2835.

[CR52] Prokop, P., M. Zvaríková, M. Zvarík, Z. Ježová, and P. Fedor. 2024. Charismatic species should be large: The role of admiration and fear. *People and Nature* 6: 945–957. 10.1002/pan3.10504.

[CR53] R Development Core Team. 2023. R: A language and environment for statistical computing. (4.3.1). R Foundation for Statistical Computing, Vienna, Austria. Available at https://cran.r-project.org/doc/manuals/r-release/fullrefman.pdf Accessed on 19 Jun 2025.

[CR54] Ribeiro, Â. M., M. Rodrigues, N. V. Brito, and T. L. Mateus. 2023. Prickly connections: Sociodemographic factors shaping attitudes, perception and biological knowledge about the European hedgehog. *Animals* 13: 3610. 10.3390/ani13233610. 38066961 10.3390/ani13233610 PMC10705511

[CR55] Sandifer, P. A., A. E. Sutton-Grier, and B. P. Ward. 2015. Exploring connections among nature, biodiversity, ecosystem services, and human health and well-being: Opportunities to enhance health and biodiversity conservation. *Ecosystem Services* 12: 1–15. 10.1016/j.ecoser.2014.12.007.

[CR56] Santini, L., M. González-Suárez, D. Russo, A. Gonzalez-Voyer, A. von Hardenberg, and L. Ancillotto. 2019. One strategy does not fit all: Determinants of urban adaptation in mammals. *Ecology Letters* 22: 365–376. 10.1111/ele.13199. 30575254 10.1111/ele.13199 PMC7379640

[CR57] Satoshi, N., T. Takahiro, O. Satoru, T. Kazuhiko, and U. Nisikawa. 2014. Exploring factors affecting farmers’ implementation of wildlife-friendly farming on Sado Island, Japan. *Journal of Resources and Ecology* 5: 370–380. 10.5814/j.issn.1674-764x.2014.04.013.

[CR58] Serangeli, M. T., L. Cistrone, L. Ancillotto, A. Tomassini, and D. Russo. 2012. The post-release fate of hand-reared orphaned bats: Survival and habitat selection. *Animal Welfare* 21: 9–18. 10.7120/096272812799129510.

[CR59] Soulsbury, C. D., and P. C. White. 2015. Human–wildlife interactions in urban areas: A review of conflicts, benefits and opportunities. *Wildlife Research* 42: 541–553. 10.1071/WR14229.

[CR60] Sweet, F. S., and W. W. Weisser. 2025. Welcome for thee, but not for me: How demographic parameters and nature experience affect how close to home people accept animals. *Basic and Applied Ecology* 87: 83–91. 10.1016/j.baae.2025.06.007.

[CR61] Sweet, F. S., P. Noack, T. E. Hauck, and W. W. Weisser. 2023. The relationship between knowing and liking for 91 urban animal species among students. *Animals* 13: 488. 10.3390/ani13030488. 36766376 10.3390/ani13030488 PMC9913501

[CR62] Sweet, F. S., A. Mimet, M. N. U. Shumon, L. P. Schirra, J. Schäffler, S. C. Haubitz, P. Noack, T. E. Hauck, et al. 2024. There is a place for every animal, but not in my back yard: a survey on attitudes towards urban animals and where people want them to live. *Journal of Urban Ecology* 10: 006. 10.1093/jue/juae006.

[CR63] Teixeira, C. P., C. O. Fernandes, R. Ryan, and J. Ahern. 2022. Attitudes and preferences towards plants in urban green spaces: Implications for the design and management of Novel Urban Ecosystems. *Journal of Environmental Management* 314: 115103. 10.1016/j.jenvman.2022.115103. 35468436 10.1016/j.jenvman.2022.115103

[CR64] Theodorou, P. 2022. The effects of urbanisation on ecological interactions. *Current Opinion in Insect Science* 52: 100922. 10.1016/j.cois.2022.100922. 35490874 10.1016/j.cois.2022.100922

[CR65] Thompson, J. L., A. Kaiser, E. L. Sparks, M. Shelton, E. Brunden, J. A. Cherry, and J. Cebrian. 2016. Ecosystem–what? Public understanding and trust in conservation science and ecosystem services. *Frontiers in Communication* 1: 3. 10.3389/fcomm.2016.00003.

[CR66] Van Dalum, M. J. 2013. *Attitude change towards wildlife and the role of environmental education Master’s thesis*. Utrecht: Utrecht University.

[CR67] Verba, S., K. L. Schlozman, H. Brady, and N. H. Nie. 1993. Citizen activity: Who participates? What do they say? *The American Political Science Review* 87: 303–318. 10.2307/2939042.

[CR68] Viviano, A., I. De Meo, E. Mori, C. Sergiacomi, and A. Paletto. 2024. Public perception and acceptance of coypu *Myocastor coypus* removal in urban areas: Influence of age and education. *The Science of Nature* 111: 42. 10.1007/s00114-024-01928-2. 10.1007/s00114-024-01928-2 PMC11297126 39093457

[CR69] Viviano, A., L. Ancillotto, O. Dondina, A. Burchielli, D. Miccolis, and E. Mori. 2025. What a camera trap survey can reveal about the behaviour of an invasive species: Insights from coypus *Myocastor coypus* in an urban park of central Italy. *Applied Animal Behaviour Science* 284: 106534. 10.1016/j.applanim.2025.106534.

[CR70] White, J., M. Kemmelmeier, S. Bassett, and J. Smith. 2018. Human perceptions of an avian predator in an urban ecosystem: Close proximity to nests increases fondness among local residents. *Urban Ecosystems* 21: 271–280. 10.1007/s11252-017-0713-y.

[CR71] Wolsko, C., and K. Lindberg. 2013. Experiencing connection with nature: The matrix of psychological well-being, mindfulness, and outdoor recreation. *Ecopsychology* 5: 80–91. 10.1089/eco.2013.0008.

[CR72] Zhou, X. H., W. Zhang, D. Y. Tang, Z. Miao, Q. Wang, and D. C. MacMillan. 2023. A quantitative analysis of public preferences for the wild boar management in urban and rural China. *Global Ecology and Conservation* 41: e02353. 10.1016/j.gecco.2022.e02353.

[CR73] Zorondo-Rodríguez, F., D. Moreira-Arce, and S. Boutin. 2020. Underlying social attitudes towards conservation of threatened carnivores in human-dominated landscapes. *Oryx* 54: 351–358. 10.1017/S0030605318000832.

