# Supplementary material for: Citizen eyes on elusive wildlife: Assessing public appreciation for urban wild mammals
Source: Ambio. 2025 Dec 9;55(7):1603–18. doi: 10.1007/s13280-025-02315-5 (PMC13230373; doi:10.1007/s13280-025-02315-5)
Supplement: Supplementary file 1 — (PDF 721 KB) [file 13280_2025_2315_MOESM1_ESM.pdf]

Supplementary Information

This Supplementary Information has not been peer reviewed

Title: **Citizen eyes on elusive wildlife: assessing public appreciation for urban wild mammals**

Emiliano Mori[1,2,*], Valentina Marchi[3,*], Olivia Dondina[2,4], Andrea Viviano[1,§], Pietro Di Bari[1,2,5], Rosario Balestrieri[6], Marida Corradetti[7], Leonardo Ancillotto[1,2]

1. Research Institute on Terrestrial Ecosystems (IRET), National Research Council of Italy (CNR), Via Madonna del Piano 10, 50019, Sesto Fiorentino (Firenze), Italy. ORCID ID: 0000-0001-8108-7950 (Emiliano Mori); 0000-0002-2970-3389 (Andrea Viviano); 0000-0001-5817-858X (Pietro Di Bari); 0000-0002-8774-0671 (Leonardo Ancillotto); Email: emiliano.mori@cnr.it; andreaviviano@cnr.it; pietro.dibari@unipa.it; leonardo.ancillotto@cnr.it
2. National Biodiversity Future Center (NBFC), Piazza Marina 61, 90133, Palermo (Palermo), Italy
3. National Research Council of Italy - Institute of BioEconomy (CNR-IBE), Via G. Caproni 8, 50145, Florence, Italy. ORCID ID: 0000-0002-0329-0707 (Valentina Marchi).
4. Department of Earth and Environmental Sciences, University of Milano-Bicocca, Piazza della Scienza 1, 20126, Milano, Italy. ORCID ID: 0000-0001-8097-1971 (Olivia Dondina).
5. Department of Earth and Marine Sciences, University of Palermo, Via Archirafi 22, 90123, Palermo (Palermo), Italy.
6. Department of Integrative Marine Ecology, Stazione Zoologica Anton Dohrn, CRIMAC, Calabria Marine Centre, Contrada Torre Spaccata, Amendolara (Cosenza), Italy. ORCID ID: 0000-0001-7245-4368 (Rosario Balestrieri).
7. Istituto Istruzione Superiore Agrario Celso Ulpiani, Via della Repubblica 30, 63100, Ascoli Piceno, Italy.

*, Equal contribution

§, corresponding author: andreaviviano@cnr.it

Citizen eyes on elusive wildlife: assessing public appreciation for urban wild mammals

Supplementary Material 1

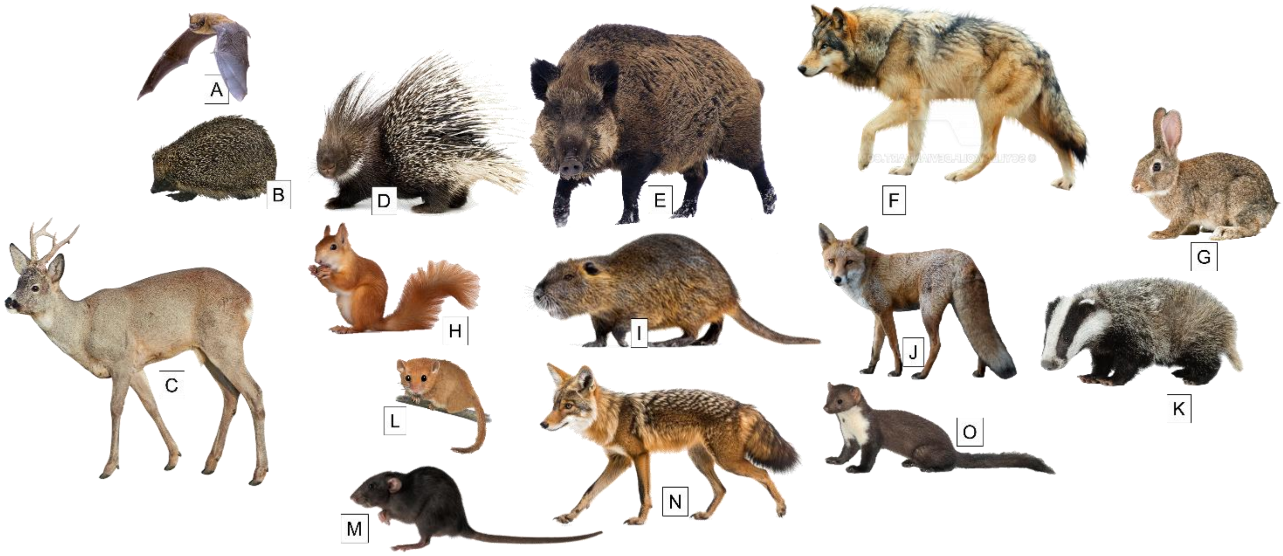

Figure S1. Coloured plate used to test for species encounter preferences by the general public. A) Kuhl’s pipistrelle; B) Hedgehog; C) Roe deer; D) Crested porcupine; E) Wild boar; F) Grey wolf; G) Rabbit; H) Red squirrel; I) Coypu; J) Red fox; K) Badger; L) Hazel dormouse; M) Black rat; N) Golden jackal; O) Stone marten.

Questionnaire

1) Are you resident in this city?

2) In which gender do you identify?

- ◻ Male
- ◻ Female
- ◻ Non-binary
- ◻ Other

3) [*Then, we showed the plate*]
The species you see on this plate are commonly found in Italian cities. Could you rank them from the one you most enjoy encountering to the one you least enjoy, by

Citizen eyes on elusive wildlife: assessing public appreciation for urban wild mammals

Supplementary Material 2

Supplementary Material 2

Table S1. Kruskal–Wallis tests.
Results of Kruskal–Wallis omnibus tests for differences in mammal perception across cities, reporting sample size (*N*), degrees of freedom (*df*), chi-square statistic (χ^2), and *p*-values.

Species	N	df	χ^2	p
Wild boar	1,000	9	313.45	< .001
G. jackal	1,000	9	304.23	< .001
Badger	1,000	9	268.68	< .001
Wolf	1,000	9	247.82	< .001
Porcupine	1,000	9	209.84	< .001
Rat	1,000	9	161.06	< .001
Hedgehog	1,000	9	101.4	< .001
Bat	1,000	9	76.56	< .001
Fox	1,000	9	65.73	< .001
Roe deer	1,000	9	59.52	< .001
Squirrel	1,000	9	42.76	< .001
Dormouse	1,000	9	41.96	< .001
Marten	1,000	9	40.2	< .001
Coypu	1,000	9	18.62	.029
Lagomorph	1,000	9	17.59	.040

Note. Sample size: Sample sizes by city: Ascoli Piceno n = 100, Empoli-Fucecchio n = 100, Firenze n = 100, Follonica n = 100, Milano n = 100, Napoli n = 100, Roma n = 100, Siracusa n = 100, Torino n = 100, Trieste n = 100.

Table S2. Dunn's post-hoc pairwise comparisons with Bonferroni correction.
Pairwise comparisons between cities for each species, reporting z statistics, unadjusted p-values, and Bonferroni-adjusted p-values. Only significant comparisons (p < .05 after Bonferroni correction) are shown.

Species	Comparison (City1 – City2)	z	p (unadj.)	p (Bonf.)
Badger	Firenze - Napoli	-12.1	< .001	< .001
Badger	Firenze - Siracusa	-11.37	< .001	< .001
Badger	Follonica - Napoli	-9.71	< .001	< .001
Badger	Ascoli Piceno - Napoli	-9.33	< .001	< .001
Badger	Follonica - Siracusa	-8.98	< .001	< .001
Badger	Ascoli Piceno - Siracusa	-8.6	< .001	< .001
Badger	Napoli - Trieste	8.49	< .001	< .001
Badger	Napoli - Roma	8.38	< .001	< .001
Badger	Siracusa - Trieste	7.77	< .001	< .001
Badger	Napoli - Torino	7.71	< .001	< .001
Badger	Roma - Siracusa	-7.65	< .001	< .001
Badger	Empoli - Napoli	-7.22	< .001	< .001

Badger	Siracusa - Torino	6.99	< .001	< .001
Badger	Firenze - Milano	-6.72	< .001	< .001
Badger	Empoli - Siracusa	-6.5	< .001	< .001
Badger	Milano - Napoli	-5.37	< .001	< .001
Badger	Empoli - Firenze	4.87	< .001	< .001
Badger	Milano - Siracusa	-4.65	< .001	< .001
Badger	Firenze - Torino	-4.38	< .001	< .001
Badger	Follonica - Milano	-4.33	< .001	< .001
Badger	Ascoli Piceno - Milano	-3.95	< .001	0.003
Badger	Firenze - Roma	-3.72	< .001	0.009
Badger	Firenze - Trieste	-3.6	< .001	0.014
Bat	Napoli - Trieste	6.45	< .001	< .001
Bat	Siracusa - Trieste	6.11	< .001	< .001
Bat	Napoli - Torino	5.36	< .001	< .001
Bat	Siracusa - Torino	5.02	< .001	< .001
Bat	Firenze - Napoli	-4.84	< .001	< .001
Bat	Firenze - Siracusa	-4.5	< .001	< .001
Bat	Milano - Trieste	4.21	< .001	0.001
Bat	Empoli - Napoli	-4.13	< .001	0.002
Bat	Napoli - Roma	3.89	< .001	0.005
Bat	Empoli - Siracusa	-3.79	< .001	0.007
Bat	Roma - Siracusa	-3.54	< .001	0.018
Bat	Ascoli Piceno - Napoli	-3.46	< .001	0.024
Bat	Follonica - Napoli	-3.4	< .001	0.030
Dormouse	Napoli - Roma	5.42	< .001	< .001
Dormouse	Empoli - Napoli	-4.96	< .001	< .001
Dormouse	Milano - Napoli	-4.7	< .001	< .001
Dormouse	Ascoli Piceno - Napoli	-4.12	< .001	0.002
Dormouse	Follonica - Napoli	-3.99	< .001	0.003
Dormouse	Napoli - Torino	3.37	< .001	0.034
Fox	Follonica - Milano	-6.27	< .001	< .001
Fox	Ascoli Piceno - Milano	-5.97	< .001	< .001
Fox	Milano - Napoli	5.41	< .001	< .001
Fox	Firenze - Milano	-5.04	< .001	< .001
Fox	Milano - Torino	4.15	< .001	0.002
Fox	Milano - Siracusa	4.06	< .001	0.002
Fox	Follonica - Trieste	-3.95	< .001	0.004
Fox	Empoli - Milano	-3.87	< .001	0.005
Fox	Follonica - Roma	-3.66	< .001	0.011
Fox	Ascoli Piceno - Trieste	-3.64	< .001	0.012
Fox	Ascoli Piceno - Roma	-3.35	< .001	0.036
Golden jackal	Napoli - Trieste	-15.75	< .001	< .001
Golden jackal	Siracusa - Trieste	-12.88	< .001	< .001
Golden jackal	Milano - Trieste	-11.26	< .001	< .001

Golden jackal	Torino - Trieste	-9.71	< .001	< .001
Golden jackal	Firenze - Trieste	-9.53	< .001	< .001
Golden jackal	Ascoli Piceno - Trieste	-8.8	< .001	< .001
Golden jackal	Follonica - Napoli	8.37	< .001	< .001
Golden jackal	Empoli - Trieste	-8.1	< .001	< .001
Golden jackal	Napoli - Roma	-7.9	< .001	< .001
Golden jackal	Roma - Trieste	-7.85	< .001	< .001
Golden jackal	Empoli - Napoli	7.65	< .001	< .001
Golden jackal	Follonica - Trieste	-7.38	< .001	< .001
Golden jackal	Ascoli Piceno - Napoli	6.95	< .001	< .001
Golden jackal	Firenze - Napoli	6.22	< .001	< .001
Golden jackal	Napoli - Torino	-6.04	< .001	< .001
Golden jackal	Follonica - Siracusa	5.49	< .001	< .001
Golden jackal	Roma - Siracusa	5.03	< .001	< .001
Golden jackal	Empoli - Siracusa	4.78	< .001	< .001
Golden jackal	Milano - Napoli	4.49	< .001	< .001
Golden jackal	Ascoli Piceno - Siracusa	4.08	< .001	0.002
Golden jackal	Follonica - Milano	3.87	< .001	0.005
Golden jackal	Milano - Roma	-3.41	< .001	0.029
Golden jackal	Firenze - Siracusa	3.34	< .001	0.037
Hedgehog	Napoli - Roma	7.42	< .001	< .001
Hedgehog	Napoli - Torino	7.03	< .001	< .001
Hedgehog	Firenze - Napoli	-6.84	< .001	< .001
Hedgehog	Empoli - Napoli	-6.82	< .001	< .001
Hedgehog	Follonica - Napoli	-6.64	< .001	< .001
Hedgehog	Ascoli Piceno - Napoli	-6.38	< .001	< .001
Hedgehog	Napoli - Trieste	5.64	< .001	< .001
Hedgehog	Milano - Napoli	-5.37	< .001	< .001
Hedgehog	Roma - Siracusa	-4.88	< .001	< .001
Hedgehog	Siracusa - Torino	4.5	< .001	< .001
Hedgehog	Firenze - Siracusa	-4.3	< .001	< .001
Hedgehog	Empoli - Siracusa	-4.29	< .001	< .001
Hedgehog	Follonica - Siracusa	-4.11	< .001	0.002
Hedgehog	Ascoli Piceno - Siracusa	-3.85	< .001	0.005
Marten	Ascoli Piceno - Milano	4.62	< .001	< .001
Marten	Milano - Torino	-4.26	< .001	< .001
Marten	Milano - Trieste	-3.94	< .001	0.004
Marten	Ascoli Piceno - Napoli	3.92	< .001	0.004
Marten	Napoli - Torino	-3.56	< .001	0.017
Marten	Follonica - Milano	3.34	< .001	0.038
Porcupine	Napoli - Trieste	10.28	< .001	< .001
Porcupine	Follonica - Napoli	-9.81	< .001	< .001
Porcupine	Ascoli Piceno - Napoli	-8.49	< .001	< .001
Porcupine	Siracusa - Trieste	8.46	< .001	< .001

Porcupine	Follonica - Siracusa	-8.0	< .001	< .001
Porcupine	Napoli - Torino	7.37	< .001	< .001
Porcupine	Firenze - Napoli	-7.19	< .001	< .001
Porcupine	Milano - Trieste	7.13	< .001	< .001
Porcupine	Empoli - Napoli	-7.0	< .001	< .001
Porcupine	Ascoli Piceno - Siracusa	-6.67	< .001	< .001
Porcupine	Follonica - Milano	-6.67	< .001	< .001
Porcupine	Napoli - Roma	5.88	< .001	< .001
Porcupine	Siracusa - Torino	5.55	< .001	< .001
Porcupine	Firenze - Siracusa	-5.38	< .001	< .001
Porcupine	Ascoli Piceno - Milano	-5.34	< .001	< .001
Porcupine	Empoli - Siracusa	-5.19	< .001	< .001
Porcupine	Roma - Trieste	4.4	< .001	< .001
Porcupine	Milano - Torino	4.22	< .001	0.001
Porcupine	Roma - Siracusa	-4.07	< .001	0.002
Porcupine	Firenze - Milano	-4.05	< .001	0.002
Porcupine	Follonica - Roma	-3.93	< .001	0.004
Porcupine	Empoli - Milano	-3.86	< .001	0.005
Porcupine	Empoli - Trieste	3.28	0.001	0.047
Rat	Firenze - Trieste	10.88	< .001	< .001
Rat	Siracusa - Trieste	9.37	< .001	< .001
Rat	Napoli - Trieste	8.62	< .001	< .001
Rat	Torino - Trieste	6.83	< .001	< .001
Rat	Follonica - Trieste	6.35	< .001	< .001
Rat	Ascoli Piceno - Firenze	-6.28	< .001	< .001
Rat	Empoli - Trieste	6.08	< .001	< .001
Rat	Firenze - Milano	5.67	< .001	< .001
Rat	Roma - Trieste	5.5	< .001	< .001
Rat	Firenze - Roma	5.38	< .001	< .001
Rat	Milano - Trieste	5.21	< .001	< .001
Rat	Empoli - Firenze	-4.8	< .001	< .001
Rat	Ascoli Piceno - Siracusa	-4.77	< .001	< .001
Rat	Ascoli Piceno - Trieste	4.6	< .001	< .001
Rat	Firenze - Follonica	4.52	< .001	< .001
Rat	Milano - Siracusa	-4.16	< .001	0.001
Rat	Firenze - Torino	4.05	< .001	0.002
Rat	Ascoli Piceno - Napoli	-4.02	< .001	0.003
Rat	Roma - Siracusa	-3.88	< .001	0.005
Rat	Milano - Napoli	-3.41	< .001	0.029
Rat	Empoli - Siracusa	-3.3	< .001	0.044
Roe deer	Siracusa - Trieste	-5.6	< .001	< .001
Roe deer	Milano - Siracusa	4.7	< .001	< .001
Roe deer	Torino - Trieste	-4.52	< .001	< .001
Roe deer	Napoli - Trieste	-4.49	< .001	< .001

Roe deer	Empoli - Siracusa	4.01	< .001	0.003
Roe deer	Follonica - Siracusa	3.85	< .001	0.005
Roe deer	Roma - Siracusa	3.79	< .001	0.007
Roe deer	Firenze - Siracusa	3.69	< .001	0.010
Roe deer	Milano - Torino	3.62	< .001	0.013
Roe deer	Ascoli Piceno - Trieste	-3.62	< .001	0.013
Roe deer	Milano - Napoli	3.59	< .001	0.015
Squirrel	Ascoli Piceno - Siracusa	4.88	< .001	< .001
Squirrel	Ascoli Piceno - Milano	4.77	< .001	< .001
Squirrel	Ascoli Piceno - Napoli	4.25	< .001	< .001
Squirrel	Ascoli Piceno - Trieste	3.63	< .001	0.013
Squirrel	Ascoli Piceno - Roma	3.35	< .001	0.036
Squirrel	Siracusa - Torino	-3.35	< .001	0.037
Wildboar	Ascoli Piceno - Siracusa	11.15	< .001	< .001
Wildboar	Ascoli Piceno - Napoli	11.04	< .001	< .001
Wildboar	Follonica - Siracusa	10.51	< .001	< .001
Wildboar	Follonica - Napoli	10.4	< .001	< .001
Wildboar	Siracusa - Torino	-9.15	< .001	< .001
Wildboar	Napoli - Torino	-9.04	< .001	< .001
Wildboar	Siracusa - Trieste	-9.03	< .001	< .001
Wildboar	Napoli - Trieste	-8.92	< .001	< .001
Wildboar	Ascoli Piceno - Milano	8.77	< .001	< .001
Wildboar	Empoli - Siracusa	8.67	< .001	< .001
Wildboar	Empoli - Napoli	8.56	< .001	< .001
Wildboar	Follonica - Milano	8.12	< .001	< .001
Wildboar	Firenze - Siracusa	7.32	< .001	< .001
Wildboar	Firenze - Napoli	7.21	< .001	< .001
Wildboar	Roma - Siracusa	7.02	< .001	< .001
Wildboar	Napoli - Roma	-6.91	< .001	< .001
Wildboar	Milano - Torino	-6.76	< .001	< .001
Wildboar	Milano - Trieste	-6.64	< .001	< .001
Wildboar	Empoli - Milano	6.28	< .001	< .001
Wildboar	Firenze - Milano	4.93	< .001	< .001
Wildboar	Milano - Roma	-4.63	< .001	< .001
Wildboar	Ascoli Piceno - Roma	4.13	< .001	0.002
Wildboar	Ascoli Piceno - Firenze	3.83	< .001	0.006
Wildboar	Follonica - Roma	3.49	< .001	0.022
Wolf	Napoli - Roma	-11.82	< .001	< .001
Wolf	Firenze - Napoli	11.77	< .001	< .001
Wolf	Napoli - Trieste	-9.99	< .001	< .001
Wolf	Empoli - Napoli	9.78	< .001	< .001
Wolf	Follonica - Napoli	9.25	< .001	< .001
Wolf	Napoli - Torino	-9.1	< .001	< .001
Wolf	Ascoli Piceno - Napoli	8.57	< .001	< .001

Wolf	Roma - Siracusa	8.26	< .001	< .001
Wolf	Firenze - Siracusa	8.21	< .001	< .001
Wolf	Milano - Napoli	6.91	< .001	< .001
Wolf	Siracusa - Trieste	-6.43	< .001	< .001
Wolf	Empoli - Siracusa	6.22	< .001	< .001
Wolf	Follonica - Siracusa	5.7	< .001	< .001
Wolf	Siracusa - Torino	-5.55	< .001	< .001
Wolf	Ascoli Piceno - Siracusa	5.01	< .001	< .001
Wolf	Milano - Roma	-4.91	< .001	< .001
Wolf	Firenze - Milano	4.86	< .001	< .001
Wolf	Napoli - Siracusa	-3.56	< .001	0.017
Wolf	Milano - Siracusa	3.35	< .001	0.036

Note. Sample size: Sample sizes by city: Ascoli Piceno n = 100, Empoli-Fucecchio n = 100, Firenze n = 100, Follonica n = 100, Milano n = 100, Napoli n = 100, Roma n = 100, Siracusa n = 100, Torino n = 100, Trieste n = 100.

Table S3. Independent samples t-tests comparing perceptions of species in cities where they are present vs. absent.

Results of Welch's t-tests examining differences in perception scores between cities where species are locally present (pre) and cities where they are absent (abs). Species occurring in all cities were excluded from the analysis, as no comparison between presence and absence was possible (rat, lagomorph, marten, bat, hedgehog, rat and fox).

Species	Npre	Nabs	Mpre	SDpre	Mabs	SDabs	t	df	p	Cohen's d
Badger	700	200	4.82	3.02	9.07	3.06	17.38	318.3	< .001	1.40
Wildboar	700	200	13.96	1.91	11.53	2.69	-11.99	259.0	< .001	-1.04
Wolf	500	400	11.25	3.44	8.29	4.60	-10.74	721.2	< .001	-0.73
G. jackal	500	400	10.23	3.41	8.65	3.85	-6.68	804.2	< .001	-0.45
Roe deer	700	200	5.87	3.05	4.71	3.21	-4.54	308.7	< .001	-0.37
Dormouse	600	300	7.69	2.96	8.53	3.49	3.58	518.4	< .001	0.26
Squirrel	700	200	5.49	3.31	4.66	3.45	-3.02	311.0	0.03	-0.24
Coypu	600	300	11.90	2.14	11.47	2.49	-2.58	525.1	0.01	-0.19
Porcupine	700	200	5.95	3.23	6.57	4.13	1.97	272.6	0.05	0.17

Note. Mpre = mean score in cities where the species is present; Mabs = mean score in cities where the species is absent. SD = standard deviation. Welch's t*-test was used; df = degrees of freedom; p=p-value. Cohen's* d *is reported as an effect size (positive values indicate higher scores in presence cities, negative values indicate higher scores in absence cities).*
